# *CHEK2* germline variants identified in familial nonmedullary thyroid cancer lead to impaired protein structure and function

**DOI:** 10.1016/j.jbc.2024.105767

**Published:** 2024-02-16

**Authors:** Carolina Pires, Inês J. Marques, Mariana Valério, Ana Saramago, Paulo E. Santo, Sandra Santos, Margarida Silva, Margarida M. Moura, João Matos, Teresa Pereira, Rafael Cabrera, Diana Lousa, Valeriano Leite, Tiago M. Bandeiras, João B. Vicente, Branca M. Cavaco

**Affiliations:** 1 Unidade de Investigação em Patobiologia Molecular, Instituto Português de Oncologia de Lisboa Francisco Gentil, Lisboa, Portugal; 2 NOVA Medical School/Faculdade de Ciências Médicas, Universidade Nova de Lisboa, Lisboa, Portugal; 3 Instituto de Tecnologia Química e Biológica António Xavier, Universidade Nova de Lisboa, Oeiras, Portugal; 4 Instituto de Biologia Experimental e Tecnológica, Oeiras, Portugal; 5 Serviço de Anatomia Patológica, Instituto Português de Oncologia de Lisboa Francisco Gentil, Lisboa, Portugal; 6 Serviço de Endocrinologia, Instituto Português de Oncologia de Lisboa Francisco Gentil, Lisboa, Portugal

**Keywords:** familial nonmedullary thyroid cancer (FNMTC), DNA repair, next-generation sequencing (NGS), *CHEK2*, molecular genetics, biophysical characterization, immunohistochemistry, molecular dynamics

## Abstract

Approximately 5 to 15% of nonmedullary thyroid cancers (NMTC) present in a familial form (familial nonmedullary thyroid cancers [FNMTC]). The genetic basis of FNMTC remains largely unknown, representing a limitation for diagnostic and clinical management. Recently, germline mutations in DNA repair-related genes have been described in cases with thyroid cancer (TC), suggesting a role in FNMTC etiology. Here, two FNMTC families were studied, each with two members affected with TC. Ninety-four hereditary cancer predisposition genes were analyzed through next-generation sequencing, revealing two germline *CHEK2* missense variants (c.962A > C, p.E321A and c.470T > C, p.I157T), which segregated with TC in each FNMTC family. p.E321A, located in the CHK2 protein kinase domain, is a rare variant, previously unreported in the literature. Conversely, p.I157T, located in CHK2 forkhead-associated domain, has been extensively described, having conflicting interpretations of pathogenicity. CHK2 proteins (WT and variants) were characterized using biophysical methods, molecular dynamics simulations, and immunohistochemistry. Overall, biophysical characterization of these CHK2 variants showed that they have compromised structural and conformational stability and impaired kinase activity, compared to the WT protein. CHK2 appears to aggregate into amyloid-like fibrils *in vitro*, which opens future perspectives toward positioning CHK2 in cancer pathophysiology. CHK2 variants exhibited higher propensity for this conformational change, also displaying higher expression in thyroid tumors. The present findings support the utility of complementary biophysical and *in silico* approaches toward understanding the impact of genetic variants in protein structure and function, improving the current knowledge on *CHEK2* variants’ role in FNMTC genetic basis, with prospective clinical translation.

Thyroid cancer (TC) is the most common endocrine malignancy, accounting for ∼3% of all cancer diagnoses worldwide (1). It continues to be the most rapidly increasing cancer type, with a male/female ratio of 1:3 (2). Most thyroid tumors (approximately 95%) are derived from the thyroid follicular cells and are designated as nonmedullary thyroid cancers (NMTC) (3). Although the majority of these tumors are sporadic, 5 to 15% of all follicular cell-derived thyroid tumors are familial, being designated as familial nonmedullary thyroid cancers (FNMTC). In FNMTC families, patients frequently present TC together with benign lesions [*e.g.*, thyroid follicular nodular disease (FND) and follicular thyroid adenomas], with papillary thyroid cancer (PTC) being the most frequent subtype (4). FNMTC can be divided into two groups based on clinical characteristics: syndromic and nonsyndromic. Despite being rare, the genetic alterations underlying syndromic FNMTC are well-defined (5). Conversely, the genetic background of nonsyndromic FNMTC and its association with clinical behavior is currently controversial and not well understood (6). Nonsyndromic FNMTC has been clinically defined in the World Health Organization classification by the presence of follicular cell-derived TC in at least three first-degree relatives, or by the existence of PTC in two or more first-degree relatives, in the absence of a history of previous radiation or inherited cancer syndrome (7). In recent years, several approaches have been undertaken to better understand FNMTC’s etiology. Genome-wide association studies, linkage analyses, targeted, and whole exome/genome sequencing led to the identification of several chromosomal *loci* [*MNG1* (14q32), *TCO* (19p13.2), *NMTC1* (2q21), *FTEN* (8p23.1-p22), *PRN* (1q21), 6q22, 8q24, and 12q14] (8), as well as predisposing risk variants in genes such as *DICER1, SRGAP1*, *NKX2*-1, *FOXE1*, *SRRM2*, *RTFC*, *HABP2*, *MYO1F, MAP2K5* and, more recently, *SPRY4* (9, 10, 11, 12, 13, 14, 15, 16, 17, 18). Additionally, germline variants in DNA repair related-genes, namely *BRCA1/2*, *ATM*, *CHEK2*, and *MSH6* have been recently reported in cases with FNMTC and NMTC (19, 20, 21, 22, 23, 24, 25), suggesting a role for these genes in FNMTC pathogenesis. Despite these findings, the molecular basis of FNMTC remains largely unknown, since these genes are only mutated in a small fraction of the families. As such, for the nonsyndromic families, genetic testing is still not covered by guidelines for diagnostic, therapeutic, and follow-up decisions (26, 27).

In this work, we used a commercial panel to analyze 94 genes associated with cancer predisposition, in two Portuguese families with FNMTC. This approach allowed the identification of two likely pathogenic variants in the *CHEK2* gene. *CHEK2* is a tumor suppressor gene that encodes the serine/threonine protein kinase CHK2, which is a key mediator of the DNA damage checkpoint that responds to DNA double-strand breaks, playing a crucial role in the maintenance of genomic integrity (28). Mutations in *CHEK2* not only exist in subsets of sporadic cancers, but they also predispose patients to several types of familial cancers (29), with increasing evidence emerging in the context of FNMTC (21, 22, 23, 24, 25). In this study, the identified CHK2 variants were structurally and functionally characterized, using biophysical methods and molecular dynamics (MD) simulations.

## Results

### Next-generation sequencing (NGS) and variant prioritization

**Figure 1:**
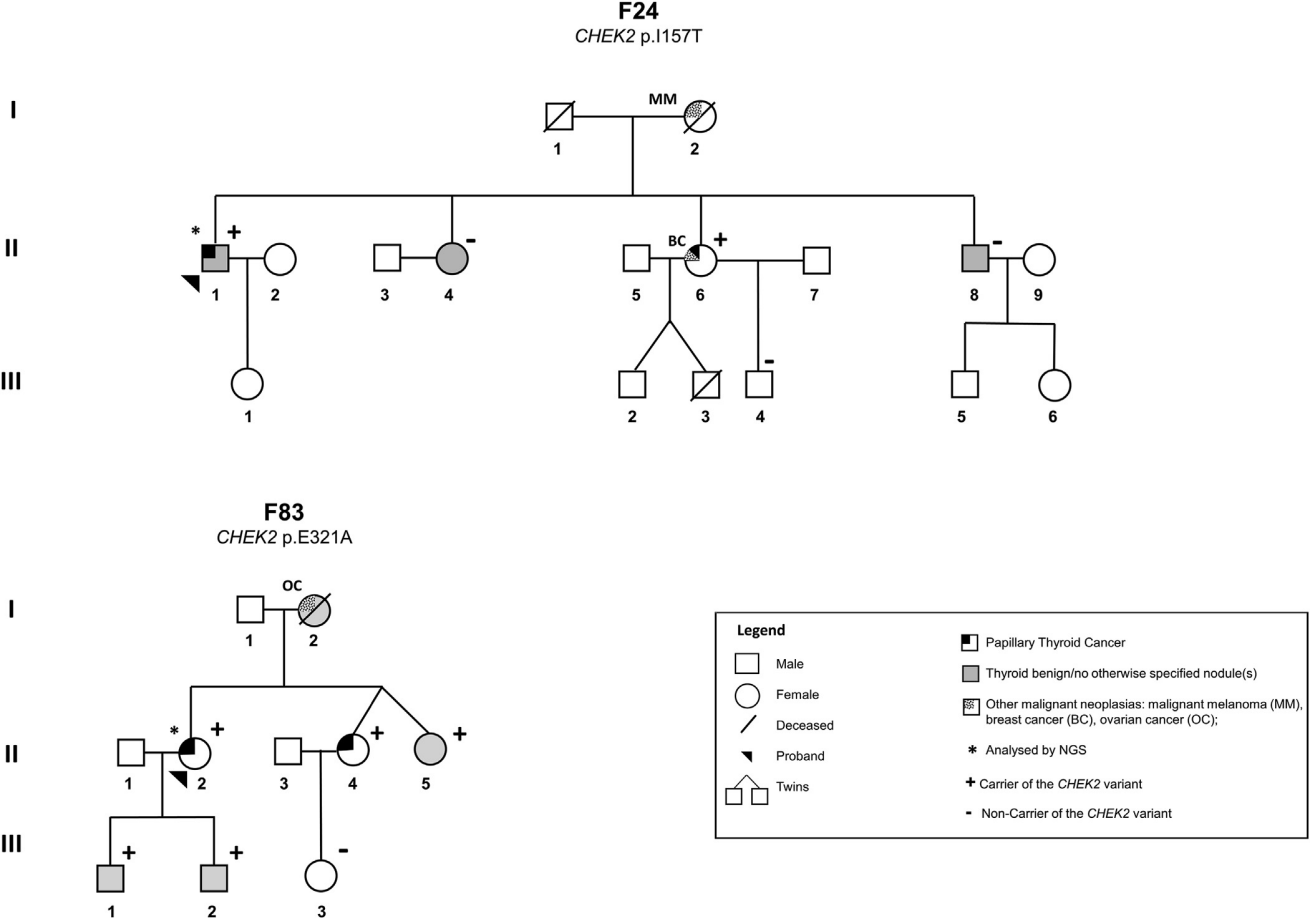
**Pedigree of the two FNMTC families with *CHEK2* variants.***CHEK2* variants p.I157T and p.E321A were identified in families F24 and F83, respectively. F, family; FNMTC, familial nonmedullary thyroid cancer.

In the present study, a screening for germline variants in 94 cancer predisposing genes was conducted in the DNA of the probands from two FNMTC families [Family 24 (F24), individual II.1; Family 83 (F83), individual II.2] (Fig. 1), through NGS analysis, using the TruSight Cancer Kit.

Following NGS, bioinformatics analysis was carried out. A total of 805 variants were detected in the two samples. Thus, in order to select the potentially pathogenic variants, specific criteria and filters were applied, revealing two germline checkpoint kinase 2 (*CHEK2*) (NM_007194.4) missense variants: c.470T > C, p.I157T (exon 4) detected in F24, and c.962A > C, p.E321A (exon 9) in F83 (NGS data bioinformatics analyses detailed in the Supporting information). Both variants were confirmed by Sanger sequencing. Segregation analysis in Family F83 showed that the *CHEK2* variant segregated with TC, thyroid FND, and no otherwise specified thyroid nodule(s) (NOS-TN) in the family. The variant was detected in heterozygosity in the proband (II.2; PTC), her two sisters [one with PTC (II.4) and one with NOS-TN (II.5)], and sons (III.1 and III.2; FND and NOS-TN, respectively). It was not possible to investigate the presence of the variant in the proband’s mother (I.2), who presented FND and ovarian cancer, since she was already deceased. In family F24, the *CHEK2* variant p.I157T segregated with TC but not with the benign/NOS thyroid lesions. The variant was found in heterozygosity in the proband (II.1) and his sister (II.6), both with PTC, but not in the siblings with FND (II.8) and a NOS-TN (II.4). Further segregation analysis was not possible due to families’ small size. Both *CHEK2* variants were absent in 100 Portuguese healthy controls.

Loss of heterozygosity (LOH) is a common genetic event in cancer development and is known to be involved in the somatic inactivation of WT alleles from tumor suppressor genes, in many inherited cancer syndromes. To investigate the second hit, the presence of LOH involving *CHEK2* was evaluated in available benign and malignant thyroid lesions from *CHEK2* variant carriers (F24: II.1 and II.6; F83: II.2 and II.4). However, no pattern suggestive of LOH was detected, as the germline variants were found in heterozygosity in the patients' tumors (data not shown). In addition, NGS analysis of the thyroid cancer (PTC) from patient II.4 from family F83 showed that there were no somatic *CHEK2* variants. This indicates that other mechanisms may be underlying tumor development in these cases (*e.g.*, loss-of-function possibly leading to haploinsufficiency or dominant-negative effects).

### *In silico* characterization of CHK2 p.I157T and p.E321A variants

The expression of the *CHEK2* gene, both at protein and mRNA level in normal thyroid gland tissues, are defined as “medium” in The Human Protein Atlas database (Table 1). The potential functional consequences of *CHEK2* candidate variants identified in this study were further investigated, adding MetaLR, Mutation Assessor and REVEL to the common set of *in silico* prediction tools (SIFT, PolyPhen, MutationTaster). Prediction of the substitutions’ effects on protein function ranged from low effect to deleterious, for the two *CHEK2* variants (Table 1). The variants are located in relevant CHK2 regions: the I157T substitution is located in the forkhead-associated (FHA) functional domain and the E321A in the kinase domain, where most pathogenic *CHEK2* variants are located (mutational hotspots) (30) (Fig. S1*A*). Both variants are present in population databases, however, p.E321A (rs374395284) is extremely rare (Table 1), whereas p.I157T (rs17879961) has a higher minor allele frequency, being reported in 0.4% of the European population. Comparative sequence analysis of CHK2 showed that amino acid E321 is highly conserved among the selected species (Fig. S1*B*), whereas the I157 position is conserved in mammals, but not in more distantly related organisms (*e.g.*, *Danio rerio*), which may suggest that a change in this amino acid may have a less relevant biological role. The tolerance of amino acid substitutions at and around positions 157 and 321 of CHK2 was predicted by SNAP2 (31) and presented as a heat-map. The isoleucine to threonine substitution (I157T), a hydrophobic to polar uncharged change, is predicted to have a neutral effect (score −23; Fig. S1*C*), while the change from a glutamic acid to alanine (E321A), a large acidic residue to a small hydrophobic one, is predicted to alter the native protein function (score +56; Fig. S1*C*). Both p.E321A and p.I157T variants are described in the ClinVar database, being respectively classified as variant of uncertain significance (VUS) (ClinVar ID: 232111) and variant with conflicting interpretations of pathogenicity (ClinVar ID: 5591), whereas the data aggregator VarSome assigned the same classification to p.E321A and classified p.I157T as likely pathogenic (Table 1). The p.I157T variant has been extensively described in the literature, in different cancer types, including breast, colon, prostate, gastric, renal, and TCs (32). However, the interpretation of these findings is conflicting, as they depend on the population (33, 34, 35, 36, 37, 38), also, the variant cosegregation with the disease in the families is variable (39), and the previous functional studies insights are not consensual (39, 40, 41, 42, 43, 44). On the other hand, the p.E321A variant has not been published in the literature, in spite of being already described in the context of hereditary breast cancer in databases (ClinVar ID: 232111) (Table 1). Altogether, compared to p.E321A, the p.I157T variant is more frequent in the population, less conserved in evolution, and already has variable and contradictory descriptions regarding its pathogenicity.

**Table 1:** *In silico* characterization of the two candidate *CHEK2* variants identified by NGS in families F24 and F83

Family	Gene (RefSeq Transcript)	Chromosomal positon (GRCh38)	Nucleotide (CDS)	Amino acid (protein)	dbSNP ID	MAF (%)	*In silico* prediction of variant effect in protein function						Annotations in databases		Expression in normal thyroid	ACMG classification Final^b^
						GnomAD/ALFA (NFE/European)	SIFT	PolyPhen	Mutation Taster	MetaLR	Mutation Assessor	REVEL	ClinVar	Varsome	mRNA/Protein^a^	
F24	***CHEK2*** (NM_007194.4)	22: 28725099	c.470T>C	p.I157T	rs17879961	0.2/0.4	Uncertain	Deleterious	Deleterious	Deleterious	Low	Uncertain	**ClinVar ID: 5591** Conflicting interpretations of pathogenicity/risk factor: Pathogenic(5); Likely pathogenic(13); Pathogenic, low penetrance(1); Established risk allele(1); Uncertain significance(9)	Likely pathogenic	Medium/Medium	Likely pathogenic
F83	***CHEK2*** (NM_007194.4)	22: 28699884	c.962A>C	p.E321A	rs374395284	0.0007/0	Uncertain	Deleterious	Deleterious	Benign	Medium	Uncertain	**ClinVar ID: 232111** Uncertain significance(9)	Uncertain significance	Medium/Medium	Likely pathogenic

Abbreviations: ACMG, American College of Medical Genetics and Genomics; CDS, coding DNA sequence; MAF, minor allele frequency; NFE, Non-Finish European.

Gene, transcript and chromosomal positions were taken from Ensembl build 38.

a The Human Protein Atlas database.

b Final ACMG classification supported by the results obtained in the present study.

In this study, in order to further clarify the role of the two missense *CHEK2* variants in the development of TC, and to extend the present knowledge beyond that already available from *in silico*, databases and literature data, we used biophysical and MD approaches for functional and structural characterization of the encoded proteins.

### Production of recombinant truncated CHK2 proteins

All recombinant truncated CHK2 proteins (according to (45); represented in Fig. S1A) were successfully purified, with yields ranging from 1.2 to 26 mg of purified monomeric protein per liter of culture. The CHK2 p.E321A variant exhibited a higher tendency to precipitate during the purification process and resulted in a significant amount of higher oligomeric forms or soluble aggregates in the preparative size exclusion chromatography (SEC), as compared to the expected functional oligomeric form (data not shown). Oligomeric profiles, monitored by analytical SEC, were similar for all proteins, exhibiting one major peak (shown in Fig. S2 for WT CHK2) corresponding to a 65 kDa-protein, ∼30% higher than the expected molecular mass for a truncated monomer. Further analyses were performed using exclusively this oligomeric form.

### Effect of variants on CHK2 kinase activity

**Figure 2:**
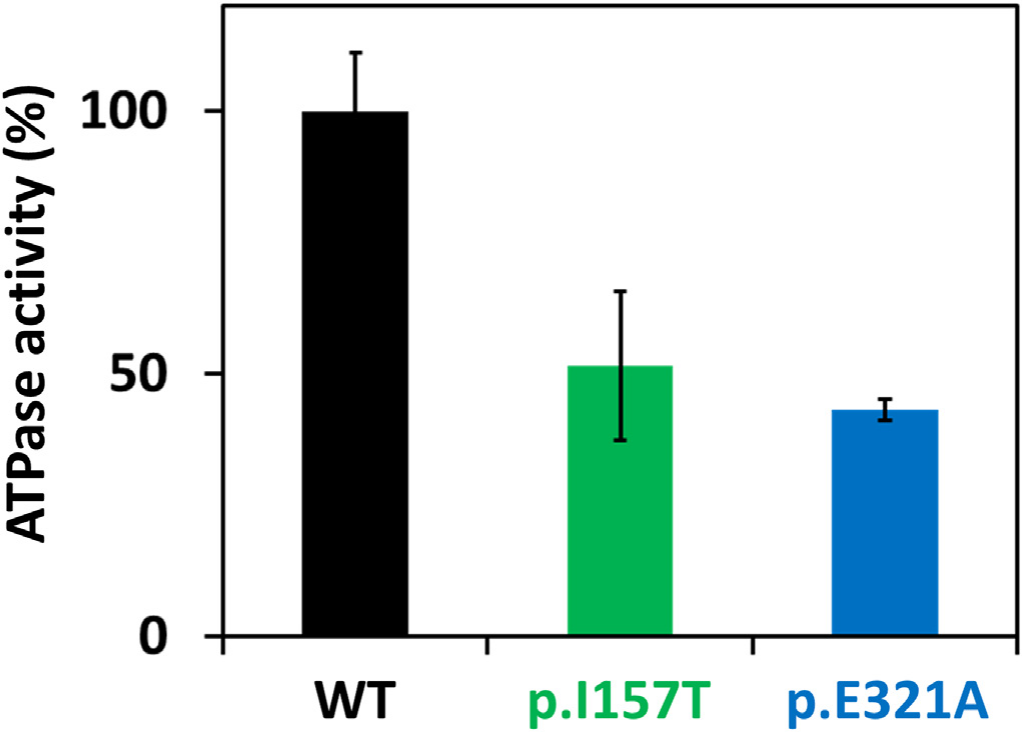
**CHK2 ATPase activity.** Enzymatic activity of CHK2 WT, p.E321A, and p.I157T was measured as relative light units (RLU). At saturating ATP, the CHK2 p.I157T and p.E321A variants presented 40 to 50% of the WT CHK2 enzymatic activity. Two independent assays were performed, in triplicate; error bars indicate the mean ± SD.

The ADP-Glo Kinase assay was performed to measure CHK2 proteins (WT, p.E321A, and p.I157T) kinase activity by quantifying the amount of ADP produced during the kinase reaction, using a peptide derived from CDC25 as the client phosphorylation target. Using equal enzyme and peptide substrate concentrations, but varying the ATP concentration, we observed that the WT protein presented a higher enzymatic activity when compared to both variants, regardless of the ATP concentration (Fig. S3). At saturating ATP, the CHK2 p.I157T and p.E321A variants presented 40 to 50% of the WT CHK2 enzymatic activity (Fig. 2).

### Effect of CHK2 variants on protein structure and stability

**Figure 3:**
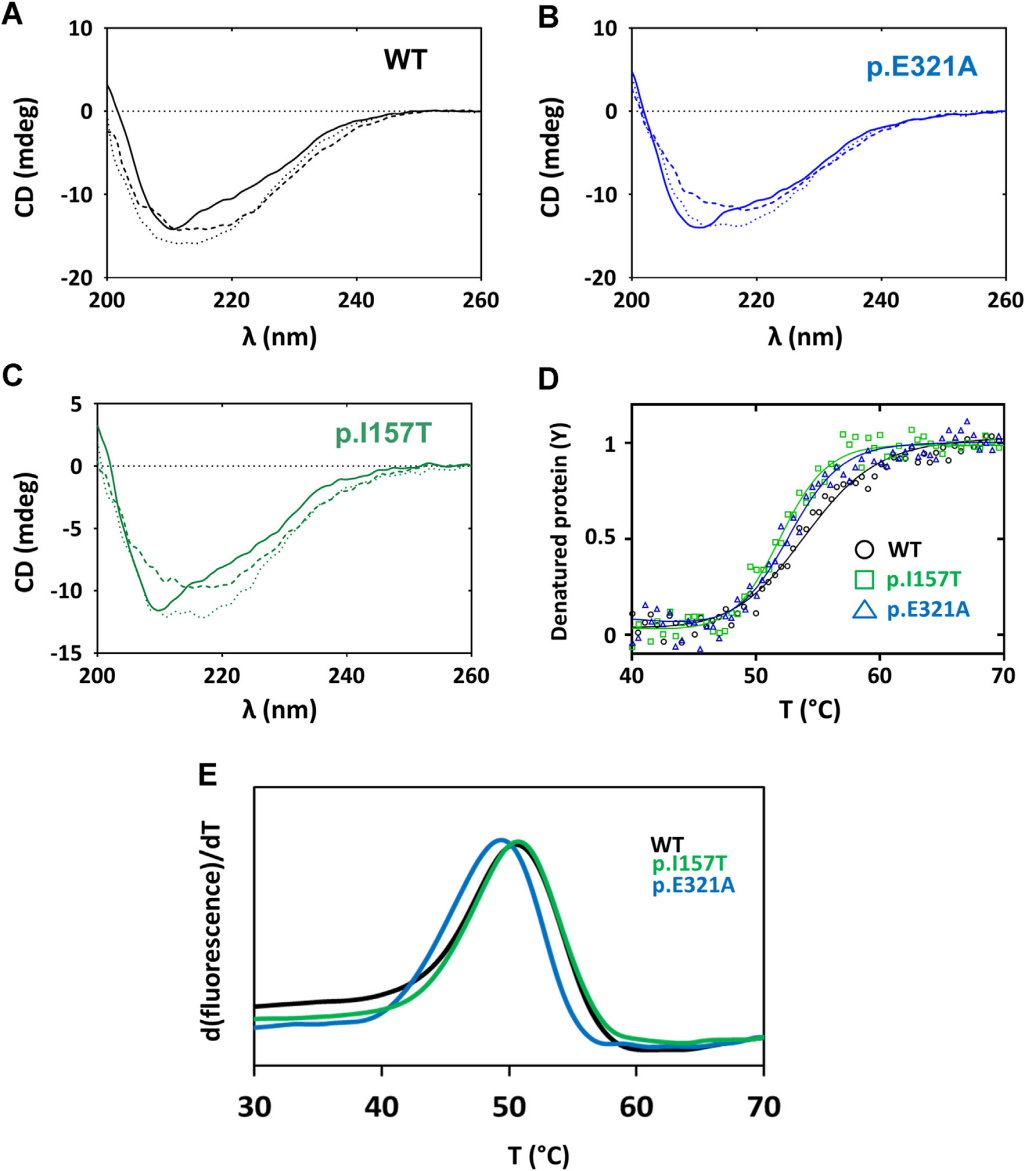
**Far-UV circular dichroism (CD) and differential scanning fluorimetry (DSF) analyses of WT and CHK2 variants.***A*–*C*, far-UV CD spectra of purified WT and CHK2 variants. Similar spectra were obtained for all proteins, indicating no significant changes in the content of secondary structure elements. *Full lines*, spectra acquired at 20 °C for each protein; *dashed lines*, spectra of fully denatured CHK2 at 90 °C; *dotted lines*, spectra upon cooling the denatured protein back to 20 °C. *D*, thermal denaturation of purified proteins obtained by monitoring molar ellipticity at 222 nm with a linear temperature increase from 20 to 90 °C at 1 °C·min^−1^, sampling 0.5 °C in a 0.1 cm light path cuvette. Replicates from two independent protein batches were performed. *E*, thermal denaturation profiles monitored by DSF were performed in a 20 μl final volume containing 0.2 mg·ml^−1^ of protein in SEC buffer and 8× Protein Thermal Shift Dye. The plates were heated from 25 to 90 °C with stepwise increments of 0.016 °C·s^−1^. Triplicates from two independent protein batches were performed. SEC, size exclusion chromatography.

CHK2 WT, p.I157T, and p.E321A exhibited similar far-UV circular dichroism (CD) spectra (Fig. 3, *A*–*C*), indicating no significant changes in the content of secondary structure elements. The obtained spectra displaying a local minimum centered at ∼210 nm and a broader band at higher λ indicated a predominantly α-helical structure with contribution from β sheets, consistent with the reported 3D structures for truncated CHK2 (45). To evaluate the effect of the substitutions on protein stability, we obtained thermal denaturation profiles by monitoring the CD at 222 nm (Fig. 3*D*). A decrease in CD at 222 nm signal to more negative values was observed, unlike what was expected for thermal unfolding of the α-helices. By taking the spectrum of fully denatured CHK2 at 90 °C, we could observe that the predominantly α-helical spectrum in native CHK2 was converted into a broad band centered at 218 nm (Fig. 3, *A*–*C*), indicative of parallel β sheets, such as those occurring in amyloid fibrils, insoluble cytotoxic protein aggregates. Upon cooling the denatured protein back to 20 °C, the spectrum remained nearly identical to the 90 °C spectrum, indicating irreversible thermal unfolding (Fig. 3, *A*–*C*). Irrespective of the final thermally denatured structure, the melting curves (presented as ascending curves for simplicity) yielded a single transition for all proteins fitted with a monophasic sigmoidal curve (Fig. 3*D*). The corresponding *T*_m_ values are presented in Table 2, with the WT protein exhibiting a slightly higher *T*_m_ value (by +1.1 to 1.7 °C) when compared to the two variants. Resistance to thermal denaturation was also evaluated by differential scanning fluorimetry (DSF) (indirect method). Thermal denaturation profiles of the three studied proteins presented a single apparent transition, in line with the CD thermal denaturation curves (Fig. 3*E*) and with no evidence for solvent exposed hydrophobic patches derived from molten globule or misfolded proteins. However, by DSF, WT CHK2 and the two variants exhibited similar *T*_m_ values (Table 2).

**Table 2:** Thermal and conformational stability and aggregation propensity of CHK2 proteins

CHK2 protein	CD	DSF	Limited proteolysis	DLS	
	Thermal denaturation			Thermal aggregation	Kinetics of isothermal aggregation
	*T*_m_ (°C)	*T*_m_ (°C)	*t*_1/2_ (min)	*T*_agg_ (°C)	*t*_1/2_ (min)
WT	53.6 ± 0.2	50.5 ± 0.1	18.8 ± 3.8	48.8 ± 1.1	49.6 ± 1.7
p.E321A	52.5 ± 0.1	49.5 ± 0.6	3.8 ± 0.9	44.8 ± 0.9	15.4 ± 0.5
p.I157T	51.9 ± 0.4	50.7 ± 0.1	6.4 ± 1.2	45.6 ± 1.2	36.4 ± 2.6

Abbreviations: CD, Far-UV circular dichroism; DLS, dynamic light scattering; DSF, differential scanning fluorimetry; T_agg_, aggregation temperature; t_1/2,_ half-life time; T_m_, melting temperature.

### Effect of CHK2 variants on conformational stability

**Figure 4:**
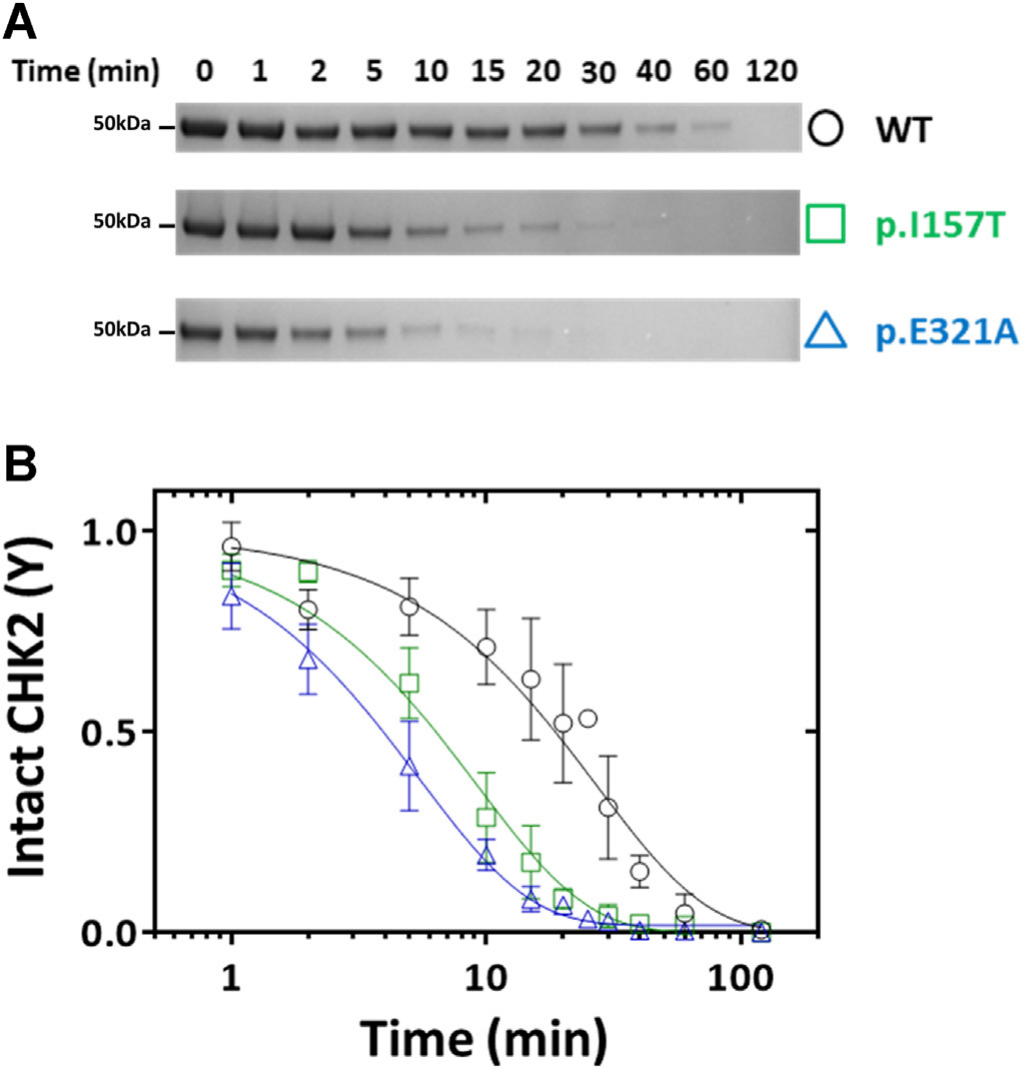
**Limited proteolysis by trypsin.***A*, representative SDS-PAGE image showing the effect of trypsin proteolysis on CHK2 WT, p.I157T, and p.E321A, at defined time points. *B*, graphic representation of the decay of intact CHK2 over time. CHK2 variants are trypsin digested at a higher proteolytic rate, particularly p.E321A. Three independent experiments were performed; error bars indicate the mean ± SD.

Limited proteolysis by trypsin was employed to assess the impact of the variants on the biologically functional conformational flexibility. Notably, while partial digestion of p.E321A was observed already at 5 min, the WT protein appeared resistant to proteolysis at least up to 20 min, when p.E321A already appeared almost completely digested (Fig. 4*A*). The p.I157T protein presented an intermediate behavior. The WT protein was only completely proteolyzed after 120 min, while in the case of p.I157T and p.E321A this occurred at around 20 to 30 min. Densitometric data corresponding to the disappearance of the full-length CHK2 band as a function of time (Fig. 4*B*) were best fitted with a single exponential decay curve, yielding a lower proteolytic rate for WT CHK2 (0.037 ± 0.006 min^−1^) than for the p.E321A (0.183 ± 0.010 min^−1^) and p.I157T (0.108 ± 0.013 min^−1^) variants. The corresponding half-lives are represented in Table 2.

### Propensity for aggregation

**Figure 5:**
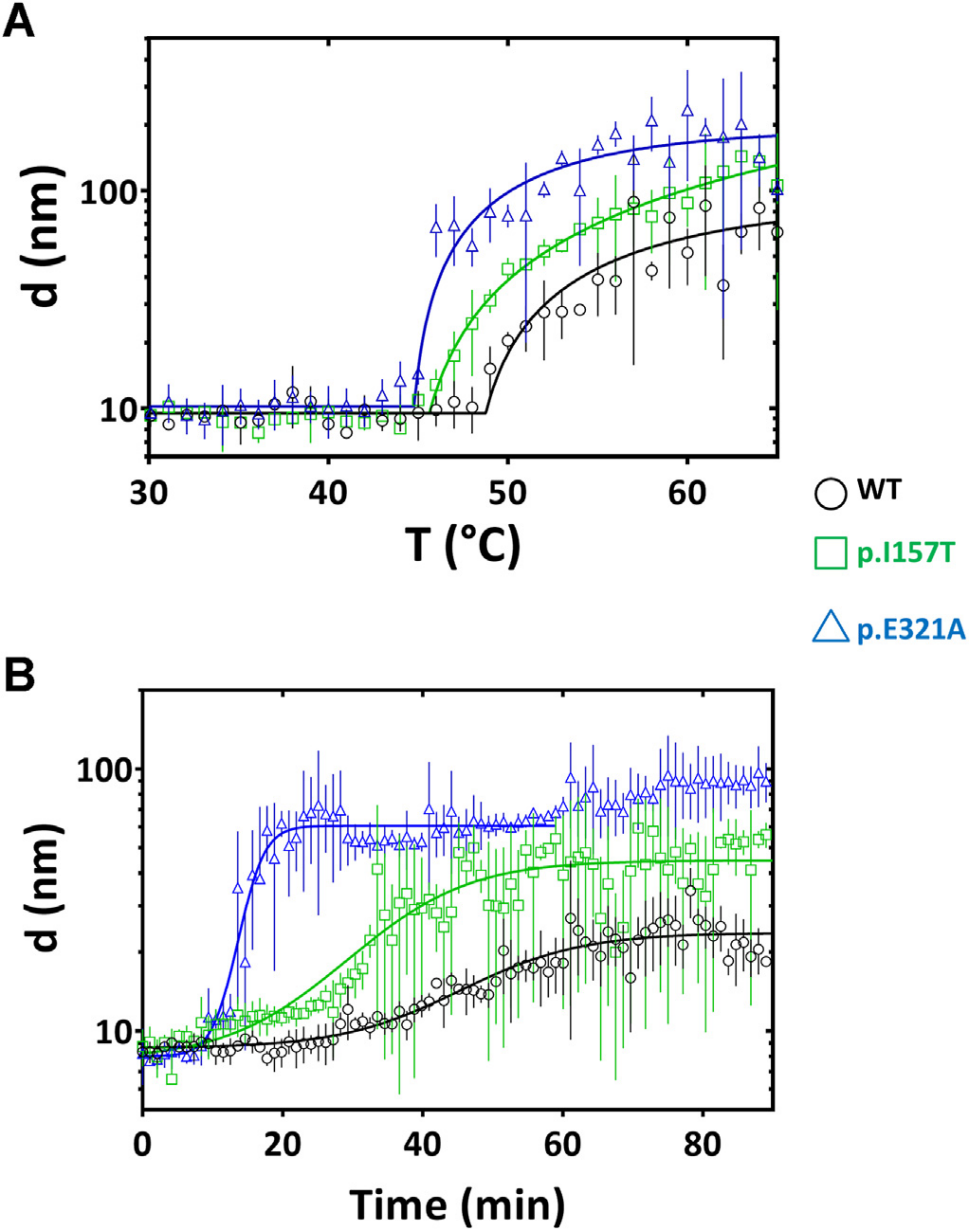
**Aggregation of WT and CHK2 variants analyzed by dynamic light scattering (DLS).***A*, thermal aggregation profile was assessed by a linear temperature increase from 20 to 90 °C at 1 °C·min^−1^. *B*, thermal aggregation kinetics followed at 42 °C during 90 min. This assay showed that p.E321A variant presents a higher propensity for aggregation and aggregates faster. Data shown are prepresentative of three independent experiments; error bars indicate the mean ± SD.

Dynamic light scattering (DLS) was used to compare the propensity of the studied proteins for aggregation. This analysis was performed as a function of linearly increasing temperature (thermal aggregation), or time, at a fixed temperature (isothermal aggregation kinetics). The particle size at resting temperature (particle size 8–9 nm) is consistent with the expected size of a flexible CHK2 monomer. Thermal aggregation profiles (Fig. 5*A*) showed that the effect of temperature on particle size remains relatively constant until a point (termed *T*_agg_) where particle size exponentially increases with temperature. As observed in Table 2, WT CHK2 has a significantly higher *T*_agg_ than the two variants (Δ*T*_agg_: +3 to 4 °C), indicating a higher propensity of the latter toward aggregation. CHK2 isothermal aggregation (Fig. 5*B*) at 42 °C revealed that at time 0 a predominant monomeric CHK2 is observed in solution. With time, an increase in size of this species was observed indicating that the protein progressively aggregates into larger species in an additive fashion. Together with the fact that the aggregation kinetics follows a sigmoidal behavior (Fig. 5*B*), this observation is consistent with the formation of fibrils. The formation of amyloid-like fibrils was further confirmed by induced CD, following the incubation of the CHK2 variants with Congo red (Fig. S4). After 1 h incubation at 42 °C in the presence of Congo red, all CHK2 variants yielded CD spectra in the visible region with the characteristic major band with λ_max_ ∼525 nm, indicative of formation of amyloid-type fibrils.

Irrespective of the aggregation mechanism, higher aggregation rates were observed for the two variants than for WT CHK2 (Table 2). In summary, both pathogenic variants display higher propensity for aggregation than WT CHK2, with p.E321A appearing as the most aggregation prone variant.

### MD simulation analysis

**Figure 6:**
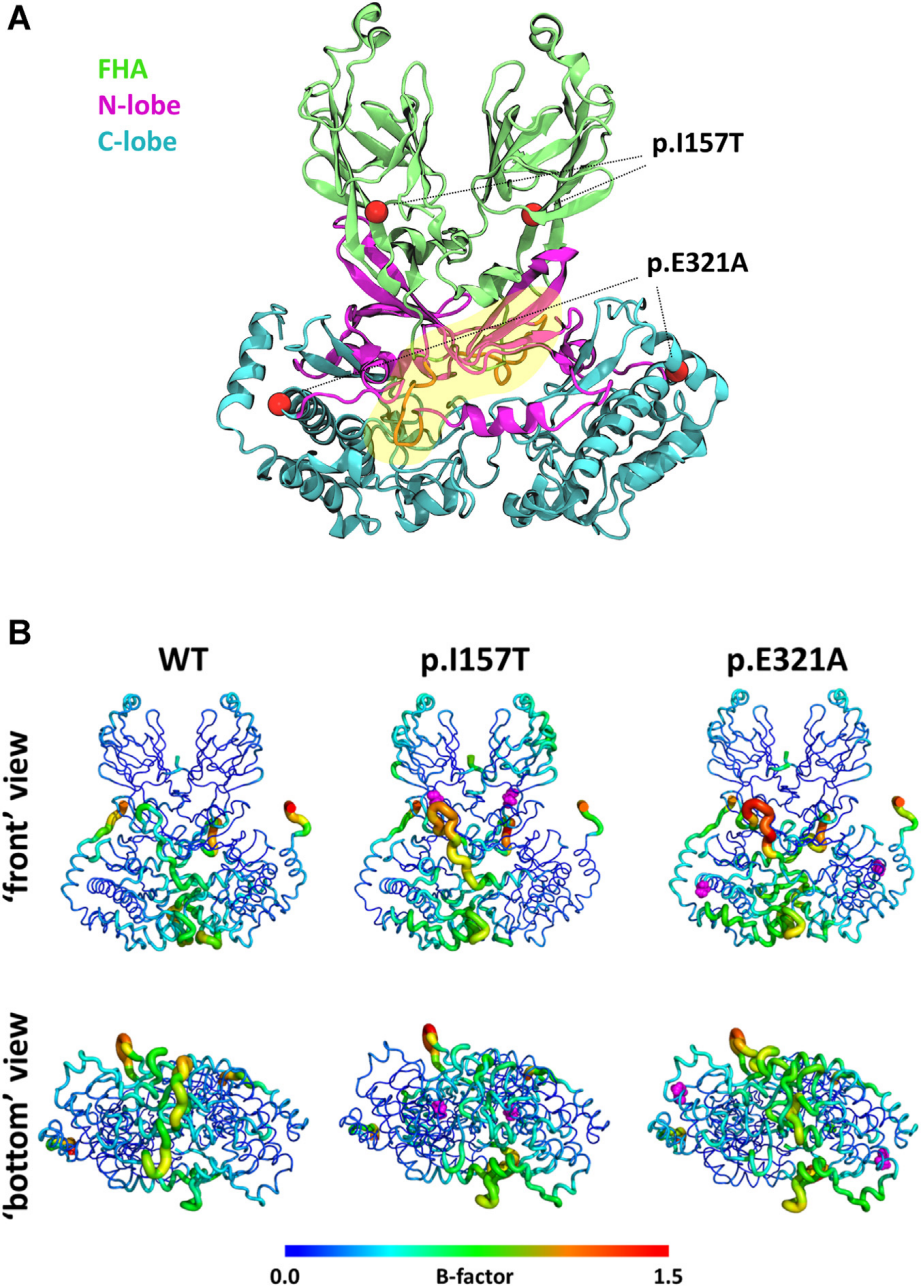
**Molecular impact of the I157T and E321A substitutions on the CHK2 dynamics predicted by molecular dynamics (MD) simulations.***A*, structure of the CHK2 dimer predicted by MODELLER v10.2, in cartoon representation. The forkhead-associated domain (FHA), N-lobe and C-lobe domains are represented in *green*, *pink* and *blue*, respectively, with the short loop between residues 258 to 266 in *orange* highlighted with a *yellow* freeform shape. The position of the amino acid substitutions (*red spheres*) in the protein variants are indicated with *dashed lines*. *B*, visualization of the B-factors based on the per-residue root-mean-square fluctuation (RMSF) of the CHK2 WT dimer and protein variants simulated. Proteins are shown using a “sausage” cartoon representation, where the thickness of the tube indicates the B-factor, reflecting the mobility of the residue. The color scale of residue mobility/flexibility goes from low (*blue*) over medium (*green*) to high (*red*). The variants’ locations are highlighted in *pink* in the respective structures.

To better understand the molecular impact of the I157T and E321A substitutions on the CHK2 dynamics, we simulated both variants and the WT protein using atomistic MD simulations (Fig. 6*A*). We started by analyzing the impact of the substitutions on the protein's flexibility by performing a root mean square fluctuation analysis for each of the residues (Fig. S5). Residue mobility data are presented in terms of B-factors. Larger B-factors are used to identify more flexible protein regions, represented in Figure 6*B* as thicker tubes and warmer colors. Results revealed that, when compared to the WT, the p.E321A variant (located in the C-lobe from the kinase domain) shows altered flexibility in the C- and N- lobes. Since the N-lobe is formed mainly by β-sheet structures (45) and a short loop between residues 258 to 266 (Fig. 6*A*), the mobility is seen mainly in the loop region. In the C-lobe, the disturbed flexibility is seen in the interface between both monomers. The FHA region does not seem to be impacted by this substitution (Fig. 6*B*). For the p.I157T variant, the increase in mobility of the loop in the N-lobe is identical to the one seen for p.E321A. The FHA region of the protein, where this substitution is located, has an increase in flexibility when compared to the WT (Fig. 6*B*). Comparing WT and the p.I157T and p.E321A variants, besides the above described affected regions at the monomer-monomer interface, there appear to be changes in flexibility on residues lining the ATP binding site, namely L226, G227, S228, G229, E273, L277, G370, and H371 (Fig. S5). Also, several predicted trypsin cleavage sites are located in regions with altered flexibility in the p.I157T and p.E321A variants as compared to WT CHK2 (Fig. S5), particularly the highly exposed K177, K253, R254, K255, R262, and R431 residues. To determine the secondary structure of each variant we performed a definition of secondary structure of proteins analysis. For all three systems the percentages of α-helical, β-sheet, coil, and bend elements are identical (Fig. S6), meaning that no significant secondary structure differences can be pinned between the variants and the WT, in agreement with far-UV CD spectra.

### Immunohistochemical analysis of CHK2 expression in patients’ thyroid lesions

To correlate with previous *in silico* and *in vitro* results, and to investigate the role of *CHEK2* p.I157T and p.E321A variants in tumor malignancy, we evaluated the expression levels of CHK2 in TC tissues and matched adjacent normal tissues from 12 patients (7 with *CHEK2* variants, including two cases with p.I157T, two cases with p.E321A, and three cases with p.Y156∗; and 5 without *CHEK2* variants) by immunohistochemistry (IHC) staining.

**Figure 7:**
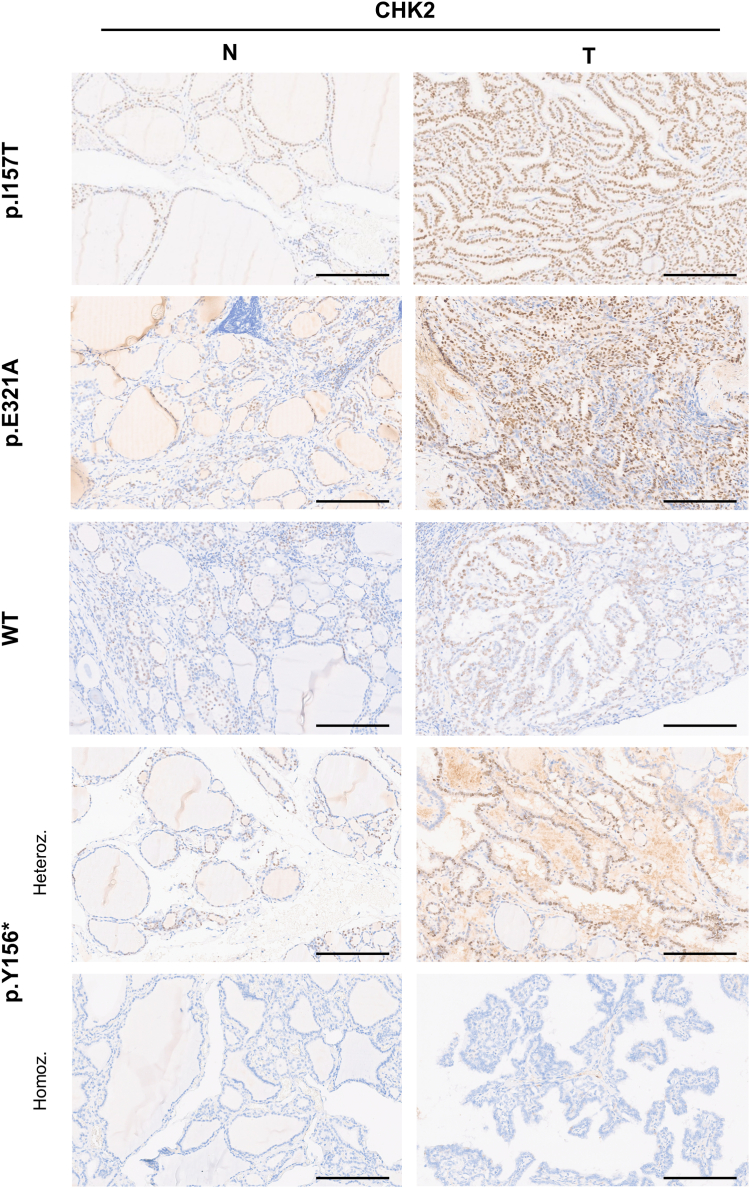
**Representative images of immunohistochemistry (IHC) staining for CHK2 in thyroid tumors and matched normal tissues.** In general, CHK2 expression was higher in the tumors (T) than in matched normal tissues (N). Tumors from patients carrying the missense germline variants p.I157T and p.E321A presented a diffuse CHK2 staining, while tumors with the germline truncating p.Y156∗ variant in heterozygosity and from WT individuals (controls without *CHEK2* variants) displayed a focal staining. The case with the p.Y156∗ variant in homozygosity was scored as negative based on the complete absence of CHK2 expression, both in tumor and normal tissues. Heteroz., heterozygous; Homoz., homozygous. Magnification 7.9×, the scale bar represents 100 μm.

Overall, all tumor and normal tissues showed positive CHK2 expression, except for one case that carried the homozygous germline truncating CHK2 p.Y156∗ variant, that completely abolished protein expression (Fig. 7 and Table S1). Moreover, CHK2 expression was increased in tumor tissues, when compared to normal tissues, independently of the presence of *CHEK2* germline variants (Fig. 7 and Table S1).

With respect to the tumors from the carriers of *CHEK2* missense variants, they more often presented a strong and diffuse CHK2 expression, since the staining was present in almost every cell nucleus, while the tumors from noncarriers of *CHEK2* variants had a focal and less strong protein expression. Furthermore, heterozygous carriers of the *CHEK2* truncating variant had a focal protein expression, contrasting with the cases carrying missense variants (Fig. 7 and Table S1).

Congo red staining was performed in the tissues from the same 12 patients and showed that amyloid deposition was undetected in both normal and tumor tissues (data not shown).

## Discussion

Recent studies reported germline variants in DNA repair genes in NMTC and FNMTC patients (19, 20, 21, 22, 23, 24, 25). In this work, we identified two distinct *CHEK2* missense variants (p.I157T and p.E321A), segregating with TC in two members from each family (F24 and F83), and with FND/NOS-TN in one family (F83). In FNMTC, multicentricity and bilaterality of thyroid carcinomas and association with hyperplastic nodules are frequently observed (7), in line with the clinicopathological findings in the present families.

*CHEK2* is a tumor suppressor gene, which encodes the serine/threonine protein kinase CHK2, a 65-kDa protein consisting of 543 amino acids, ubiquitously expressed in normal cells and tissues (46). CHK2 kinase is a key component of the DNA damage response, also participating in several molecular processes involved in DNA structure modification and cell cycle progression (28). *CHEK2* mutations are known to increase breast, ovarian, thyroid, pancreas, colon, prostate, and kidney cancer risk, being considered a multiorgan cancer susceptibility gene (29), with moderate penetrance (47). Interestingly, both families included in this study, although having the thyroid as leading affected tissue, also had members with other neoplasia in breast and ovary, and malignant melanoma.

The *CHEK2* missense p.I157T variant found in F24 has been widely studied and discussed in the literature. In TC in general (not specified for familial/sporadic status), this substitution was associated with an almost two-fold increase in the risk of PTC in the Great Poland population, and, in homozygous women, this risk is increased almost 13-fold (48). Additionally, Siołek *et al.* (49) found that carriers of the p.I157T *CHEK2* variant more frequently presented family history of TC. However, the interpretations of this variant’s pathogenicity are not consensual (ClinVar ID: 5591). One of the reasons is for being detected at frequencies above 1% in certain populations, likely due to founder effects (29, 50). Moreover, some studies indicated lack of risk association with ovarian or hereditary breast cancers (50, 51). Unlike p.I157T, p.E321A variant has no reports in the literature, having only been described as VUS (ClinVar ID: 232111).

VUS currently account for more than half of germline *CHEK2* variants reported in ClinVar, which is a bottleneck limiting the clinical translation of NGS analyses (32). Therefore, a systematic analysis of *CHEK2* VUS is crucial. Thus, in the present study, both p.I157T and p.E321A proteins were functionally and structurally characterized, using a combination of advanced *in silico* (molecular dynamics) and *in vitro* biophysical integrated approaches.

CHK2 function is linked to its ability to assemble as a homodimer, each monomer comprising three conserved functional domains: a predictably highly disordered SQ/TQ cluster domain (residues 19–69), an FHA domain (residues 92–205), and a kinase domain (residues 213–501) (45). The FHA domain, where p.I157T is located, is arranged in an 11-stranded β sandwich, being responsible for the interactions with phosphorylated proteins, including the phosphorylated SQ/TQ cluster domain of other CHK2 molecules (52). The kinase domain, location of p.E321A, consists of an N-terminal lobe (residues 213–305), formed mainly by β-sheet structures, and a larger C-terminal lobe (306–501), mostly α-helical, forming an ATP-binding site at the cleft between them (45). Here, we produced and purified three recombinant truncated monomeric CHK2 proteins, consisting of residues 70 to 512 (45): WT, p.I157T, and p.E321A.

In response to DNA damage, CHK2 is activated and in turn phosphorylates multiple client protein targets involved in DNA repair, cell cycle regulation, p53 signaling, and apoptosis (*e.g.*, CDC25A, CDC25C, E2F1, p53, BRCA1, and PML) (28). We thus employed an enzymatic assay to evaluate ATP-dependent phosphorylation of a CDC25-derived peptide substrate and observed a moderate impact of both substitutions on CHK2 activity, at a saturating ATP concentration. This observation is in accordance with the MD simulations prediction of disturbed flexibility in the N-lobe and, in particular, in several residues that line the ATP binding site, for the two variants. Previous studies on p.I157T CHK2 demonstrated lower kinase activity and reduced binding to downstream targets *in vitro*, and impaired capacity of dimerization and autophosphorylation *in vivo* (39, 41, 42). On the other hand, a single study evaluating GST-tagged p.I157T CHK2, produced in insect cells, reported a similar behavior to the WT protein (53). As regards to the rare p.E321A variant, notably, this is located at an amino acid residue where another missense change has been identified (p.E321K) and shown to abolish CHK2 kinase activity *in vitro* (54).

The moderate functional impairment prompted us to analyze in depth the variants’ structural impact in terms of protein stability and aggregation, with respect to WT CHK2. The CD spectra for WT and CHK2 variants were consistent with the secondary structure in the reported crystallographic structures. Moreover, the MD simulations also predicted absent relevant secondary structure changes resulting from the amino acid substitutions. The resistance to thermal denaturation, probed both by CD and DSF, also revealed an overall similar behavior of the variants with respect to WT CHK2. This is consistent with previously reported data showing similar stability for WT and p.I157T CHK2, ectopically expressed in U-2-OS cells (33). Unlike the thermal stability, CHK2 variants displayed a markedly impaired conformational stability. Limited proteolysis by trypsin showed a ∼3 × and ∼5 × decrease in half-life, respectively, of p.I157T and p.E321A, compared to WT CHK2. Approximately 28% of the predicted trypsin cleavage sites in CHK2 are found in the N-lobe and 36% in the C-lobe (55). According to the MD simulations, particular trypsin cleavage sites are predicted to have higher flexibility in both CHK2 variants with respect to WT, facilitating the exposure of CHK2 to solvent, and consequently to trypsin digestion. Both limited proteolysis and MD simulations thus neatly converged toward assigning an increased flexibility of specific regions in the CHK2 variants to the increased susceptibility to proteolysis. The dimerization of CHK2 is a critical event for its function *in vivo*, which is also likely to be affected by disturbed flexibility of particular regions of the protein, especially those at the monomer-monomer interface. Indeed, several patches spread across the monomer-monomer interface were predicted by MD simulations to present disturbed flexibility and, consequently, diminished stability. Accordingly, a higher propensity toward aggregation was observed by DLS. From the resting temperature, each CHK2 variant exhibited a DLS profile consistent with a predominant monomeric species. Instead of a gradual disappearance of the monomeric species concurrent with the appearance of larger aggregates, we observed a progressive increase in size of the abundant initially monomeric particle. This growing behavior is suggestive of the formation of amyloid-like fibrils, consistent with the CD spectra obtained for the 90 °C thermally denatured CHK2 (WT and variants). Interestingly, the formation of amyloid-like fibrils was further confirmed by induced CD in the presence of Congo red at 42 °C. Irrespective of the aggregation mechanism, both variants aggregated faster and at a lower temperature than the WT.

Whereas an increased propensity for aggregation may lead to a decreased stability and protein depletion due to degradation, the possibility that CHK2 aggregates into amyloid-like fibrils may actually result in protein accumulation. Congo red stain is the gold standard for the demonstration of amyloid in tissue sections. In the present study, we were unable to detect amyloid deposits in Congo red stained tissues from the *CHEK2* mutation carriers, collected in physiological conditions. We hypothesize that, in such conditions, the formation of amyloid-like fibrils may not be sufficient for their detection as amyloid plaques. Indeed, as far as we know, no study has provided evidence of amyloid aggregates associated with CHK2, nor in malignant follicular cell-derived thyroid tumors.

Here, immunohistochemical analysis revealed that tumor tissues had higher CHK2 protein levels compared to the adjacent normal tissues, regardless of the *CHEK2* mutation status of the patients, which appears to be a mechanism of the cells to signalize a defect in the DNA damage response pathway (56). Furthermore, the tumors from F24 and F83 family members, harboring the *CHEK2* missense variants, tended to present a stronger and more diffuse CHK2 expression than tumors from those without *CHEK2* mutations. Noteworthy, no evidence of a second hit was found in *CHEK2* variant carriers. Accordingly, immunohistochemical studies developed by others showed that tumors harboring the *CHEK2* p.I157T substitution had a high level of CHK2 expression (33). Interestingly, these data resemble the known correlation between *TP53* mutations and the immunohistochemical staining patterns of p53 in several cancer types (57, 58). Strong and diffuse immunodetection of p53 generally indicates a missense *TP53* mutation, while weak expression generally correlates with WT *TP53* (58).

Overall, CHK2 protein variants p.I157T and p.E321A presented reduced conformational stability, higher propensity for aggregation, and reduced kinase activity, when compared to the WT. These features were more significant for the rare variant, p.E321A, and for both cases, may indicate protein loss of function possibly leading to haploinsufficiency. Still, the observation that the CHK2 variants aggregated with enhanced propensity into amyloid-like fibrils *in vitro*, displaying high expression in tumor samples, could be related to dominant-negative effects, whereby the WT protein is prevented from carrying out its function by binding to the variant protein, leading to its accumulation and consequent disproportionate loss of function. Others have observed that the CHK2 WT-I157T heterodimer undermines the function of normal CHK2 when coexpressed in cultured human cells (33). Thus, heterozygosity for these CHK2 variants may significantly reduce the overall functional pool of CHK2 even without loss of the second, WT allele, a hypothesis that is consistent with our immunohistochemical findings. However, the possibility that this amyloid-like aggregation may be cytotoxic and/or elicit other cancer-related mechanisms cannot be excluded. Such behavior has been reported for p53, where amyloid forms of this protein have been shown to result in the loss of tumor suppressive functions and gain of tumorigenic properties, resulting in metabolic adaptations and effects on various signaling pathways (59, 60). To our knowledge, this is the first report of CHK2 aggregating into fibrils *in vitro*, which opens future perspectives toward positioning CHK2 in cancer pathophysiology.

Our finding that apparently discrete amino acid substitutions can propagate to different regions of the protein and disturb its conformation, in such a way to impair enzyme function and promote protein aggregation into fibrils, supports the relevance of biophysical and MD studies as complementary approaches toward understanding the molecular basis and impact of each genetic variant.

Due to the small size of the families, the identification of a larger number of familial TC cases with these *CHEK2* alterations is warranted to further evaluate and confirm their cosegregation with disease. Moreover, additional unidentified genetic alterations in the present families may have also contributed to disease manifestation.

Taking together, the herein described *in silico* and *in vitro* evidence may support variants’ p.I157T and p.E321A reclassification as likely pathogenic, according to American College of Medical Genetics and Genomics guidelines (61), with impact in patient management, and reinforced the role of *CHEK2* in familial TC predisposition.

## Experimental procedures

### Ethics statement

This study was approved by the Ethics Committee of Instituto Português de Oncologia de Lisboa Francisco Gentil (IPOLFG), and is in accordance with the ethical standards from the Declaration of Helsinki and its later amendments or comparable ethical standards. The collection of biological samples from all subjects involved in this study was performed after written informed consent. This article does not contain any studies with animals performed by any of the authors.

### Patients

Two FNMTC families from our cohort, already reported (62), followed in the Endocrinology Department from IPOLFG, were analyzed in the present study. Both family 24 (F24) and family 83 (F83) had two members affected with NMTC, members with benign thyroid lesions, and some members presented additional neoplasia, as follows: F24 had two members with PTC [II.1 (bilateral multifocal classic PTC with tall cells and oncocytic areas, with lymph node metastases, and FND), II.6 (bilateral multifocal classic PTC, with lymph node metastases; breast cancer)], two members with thyroid benign/no otherwise specified nodule(s) (II.8, II.4), and one member died due to a malignant melanoma (I.2); F83 had two members with PTC [II.2 (bilateral multicentric PTC, with lymph node metastases and lymphocytic thyroiditis), II.4 (multifocal classic PTC with oncocytic areas, and lymphocytic thyroiditis)], and four members affected with thyroid benign/no otherwise specified nodule(s) (I.2, II.5, III.1, and III.2), one of these, already deceased (I.2), also had ovarian cancer (Fig. 1). The histological classification of the tumors was done according to the prevailing criteria at the time of diagnosis.

DNAs from peripheral blood leukocytes from eight affected and unaffected family members, and 12 formalin-fixed paraffin-embedded (FFPE) samples from familial cases were available for study. A total of 100 DNA samples of peripheral blood leukocytes from healthy controls were also used (60% females and 40% males; median age 64 years, samples supplied by Biobanco-iMM, Lisbon Academic Medical Center, Lisbon, Portugal).

### DNA extraction

The details of DNA extraction are described in the Supporting information.

### Next-generation sequencing

The leukocyte DNAs of the probands from two FNMTC families (F24, II.1; and F83, II.2) were selected. For NGS analysis, a multigene panel (TruSight Cancer Panel, Illumina) was used as an enrichment system targeted to exons (and splice site regions) of 94 specific genes. Libraries were subjected to cluster generation on flow cell and paired-end sequencing in a MiSeq sequencer platform (Illumina). Sequence data were analyzed with the instrument software MiSeq Reporter v.2.5.1 (Illumina https://www.illumina.com/systems/sequencing-platforms/miseq/products-services/miseq-reporter.html), and the reads were aligned against the human reference sequence GRCh37. The resulting VCF files were visualized using the VariantStudio v.3 software (https://support.illumina.com/downloads/variantstudio-software.html; Illumina), which supplies the report of all detected sequence variants and the respective annotation.

### Polymerase chain reaction

PCR was performed using Taq DNA polymerase (Invitrogen) protocol. Specific primers were designed for validation and segregation analyses of the genetic variants under study.

### Sanger sequencing and mutation analysis

Sanger sequencing was used to validate and analyze segregation of the variants identified by NGS in the corresponding families. Details are described in the Supporting information.

### Immunohistochemistry

Immunohistochemical detection of CHK2 protein was performed on archival FFPE tumor samples. A total of 12 familial cases, assessed for *CHEK2* germline mutational status, were included: two from F24 (p.I157T carriers), two from F83 (p.E321A carriers), three *CHEK2* p.Y156∗ carriers [described in a previous study (23)], and the remaining were *CHEK2*-negative patients. The adjacent normal tissue in each tumor sample was analyzed as control. IHC was performed on Ventana Benchmark ULTRA equipment (Roche Diagnostics) with the Ventana Optiview DAB IHC detection kit (ref 760-700; Roche Diagnostics). CC2 was used for antigen retrieval, for 40 min, at 95 °C and a primary recombinant antibody against CHK2 protein (ab109413, Abcam) was used at 1:200 dilution and incubated for 32 min. This antibody has been KO validated by the manufacturer, *via* CRISPR-Cas9 genome editing, to confirm antibody specificity. CHK2 IHC results were interpreted in terms of expression (positive or negative), staining intensity (strong, moderate, or weak), and extent (focal or diffuse).

### Protein expression and purification

Herein, recombinant CHK2 was produced as a truncated version, comprising residues 70 to 512 [crystallographic structure available in the Protein Data Bank (PDB): 3I6W], to minimize protein instability associated with highly flexible N-terminal and C-terminal regions, according to (45). The synthetic gene, codon optimized for expression in *Escherichia coli*, was cloned into pET28a (ProteoGenix). For optimization of protein production, cell growth conditions were tested regarding *E. coli* host strain and growth media. Protein purity was analyzed by SDS-PAGE, and the protein concentration determined by the Bradford assay or NanoDrop (Thermo Fisher Scientific). Pure proteins were aliquoted, flash frozen in liquid nitrogen, and stored at −80 °C until further usage. Further details regarding protein expression and purification are described in the Supporting information.

### CHK2 kinase activity assays

CHK2 kinase activity was assessed for WT, p.E321A, and p.I157T, using the ADP-Glo Kinase Assay (Promega), according to the manufacturer’s protocol, with minor modifications. Details are described in the Supporting information.

### Far-UV circular dichroism

Far-UV CD spectra and thermal denaturation profiles were recorded in a Jasco J-815 spectropolarimeter (Easton) equipped with a Jasco CDF-426S Peltier temperature controller, in a 0.1 cm path quartz cuvette. Samples were diluted to 0.15 mg·ml^−1^ in SEC buffer. Spectra were acquired at 20 °C as follows: eight accumulations; 50 nm·min^−1^ scan rate; data pitch, 0.5 nm; data integration time, 1 s; bandwidth, 1 nm; N_2_ flow, 8 l·min^−1^. Thermal denaturation profiles were obtained by monitoring molar ellipticity at 222 nm, in the 20 to 90 °C temperature range (1 °C·min^−1^ linear temperature gradient; data pitch, 0.5 °C; data integration time, 1 s; bandwidth, 1 nm; N_2_ flow, 4 l·min^−1^). Thermal denaturation curves were analyzed according to a two-state model (least squares method).

Formation of amyloid-like fibrils at 42 °C was analyzed by induced CD resulting from reaction of fibrils with Congo red. Experiments were carried out in a dual chamber quartz cuvette, each chamber with a 4.38 mm optical path (Hellma Analytics). CHK2 variants at 0.2 mg·ml^−1^ in SEC buffer were placed in one chamber, while 80 μM Congo red (Sigma-Aldrich) in SEC buffer was placed in the other chamber. The cuvette was moved to the holder and a spectrum at time 0 was recorded. After 2 min, the cuvette was inverted several times to ensure mixing of the two solutions in the headspace of the cuvette and put back to the holder. Spectra were recorded at time intervals up to 1 h. Spectra were recorded at 200 nm·min^−1^ between 650 and 300 nm (data pitch, 0.5 nm; data integration time, 2 s; N_2_ flow, 4 l·min^−1^).

### Differential scanning fluorimetry

DSF assays were performed in a QuantStudio 7 Flex Real-Time PCR System (Thermo Fisher Scientific). CHK2 proteins prepared to a final concentration of 4 mg·ml^−1^ in SEC buffer were mixed with Protein Thermal Shift Dye (Thermo Fisher Scientific) at an 8 × working concentration, in a 20 μl total reaction volume. The plates were heated from 25 to 90 °C with stepwise increments of 0.016 °C·s^−1^, followed by the fluorescence read-out at excitation and emission wavelengths of 580 and 623 nm, respectively. Thermal denaturation curves were fitted to a monophasic sigmoidal function, with the *T*_m_ values representing the inflexion points of each transition.

### Dynamic light scattering

DLS data were acquired in a ZetaSizer Nano-S (Malvern Instruments) particle size analyzer, coupled to a Peltier temperature control unit, using a He-Ne laser as light source (633 nm). Samples were diluted in SEC buffer to 0.15 mg·ml^−1^ and centrifuged at 10,000*g* for 4 min at 4 °C to remove large aggregates. Thermal aggregation profiles were obtained between 20 and 90 °C at 1 °C·min^−1^, recording particle size average, distribution, and total scattering intensity. Data were processed using Zetasizer Nano DTS software v 7.12 (https://www.malvernpanalytical.com/en/support/product-support/software/zetasizer-family-software-update-v7-12; Malvern Instrument). Data were analyzed by plotting particle size as a function of temperature, and the aggregation (https://www.malvernpanalytical.com/en/support/product-support/software/zetasizer-family-software-update-v7-12) temperature (T_agg_) was defined as the onset of particle size increase. Aggregation kinetics was studied by monitoring particle size increase at 42 °C, over time (for approximately 90 min).

### Limited proteolysis by trypsin

Purified CHK2 WT, p.E321A, and p.I157T proteins were diluted to 0.2 mg·ml^−1^ and incubated with trypsin (Sigma-Aldrich) at a 1:200 ratio (m/m), at 37 °C for 120 min. At defined time intervals, the proteolysis reaction was quenched by addition of SDS-PAGE loading buffer and formic acid. Samples were analyzed by SDS-PAGE. The intensity of the gel bands, particularly those corresponding to intact CHK2, was determined by densitometric analysis using ImageJ (v.1.53a, National Institutes of Health, USA https://imagej.net/ij/). The results were fitted to a mono-exponential decay curve to obtain the observed proteolytic rate constant *k*_obs_.

### MD simulations

MD simulations were performed for the WT and both CHK2 variants (p.I157T and p.E321A). System preparation was identical for all variants: (i) experimental structures of the truncated proteins were obtained from PDB (3I6W); (ii) all missing residues and/or atoms in the PDB files were added using MODELLER v10.2 (63); (iii) overlapping side chains were fixed with Scwrl; and (iv) substitutions I157T and E321A were generated using PyMOL (http://www.Pymol.org/). Details are described in the Supporting information.

### Congo red staining of thyroid tissues

Amyloid deposition in the thyroid tumors and adjacent normal tissues was investigated in 5 μm sections from FFPE tissues of the cases included in the immunohistochemistry analysis, by Congo red staining. Apple-green birefringence after Congo red staining under polarized light is indicative for the presence of amyloid. Congo red-stained slides were observed using an Olympus BX40F4 light microscope (Olympus).

### Statistical analysis

For kinase activity, CD and DSF, two independent assays were performed, in triplicate, using two independent protein batches. For limited proteolysis and DLS, data shown are representatives of three independent experiments. The results are expressed as the mean ± standard deviation.

## Data availability

The data generated during and/or analyzed during the current study are available from the corresponding author on reasonable request.

## Supporting information

This article contains Supporting information (64, 65, 66, 67, 68, 69, 70, 71, 72, 73).

## Conflict of interest

The authors declare that they have no conflicts of interest with the contents of this article.
