# Supplementary material for: CHEK2 germline variants identified in familial nonmedullary thyroid cancer lead to impaired protein structure and function
Source: J Biol Chem. 2024 Feb 16;300(3):105767. doi: 10.1016/j.jbc.2024.105767 (PMC10956065; doi:10.1016/j.jbc.2024.105767)
Supplement: Supporting information [file mmc1.pdf]

Supporting information

CHEK2 germline variants identified in familial nonmedullary thyroid cancer lead to impaired protein structure and function

Carolina Pires, Inês J. Marques, Mariana Valério, Ana Saramago, Paulo E. Santo, Sandra Santos, Margarida Silva, Margarida M. Moura, João Matos, Teresa Pereira, Rafael Cabrera, Diana Lousa, Valeriano Leite, Tiago M. Bandeiras, João B. Vicente, Branca M. Cavaco

Table of contents

1. Supporting Experimental procedures

2. Supporting Figures

3. Supporting Tables

Experimental procedures

DNA extraction

The biological samples analyzed consisted of blood and formalin-fixed paraffin-embedded (FFPE) samples of normal and thyroid tumor tissues. Leukocyte DNA was extracted and purified using the Puregene® Blood Core Kit (Qiagen, Hilden, Germany), according to the manufacturer's protocol. Extracted DNA was quantified by UV spectrophotometry (NanoDrop 2000, Thermo Fisher Scientific, Wilmington, DE, USA). DNA from normal thyroid and tumor FFPE tissues was extracted using Maxwell® RSC DNA FFPE Kit (Promega, Madison, WI, USA), according to the manufacturer's protocol with minor alterations, and integrity was evaluated by agarose gel electrophoresis.

Bioinformatics analysis of next-generation sequencing (NGS) data

The leukocyte DNAs of the probands from two FNMTC families (F24, II.1; and F83, II.2) were selected. For next-generation sequencing (NGS) analysis, a multigene panel (TruSight Cancer Panel, Illumina, San Diego, USA) was used as an enrichment system targeted to exons (and splice site regions) of 94 specific genes. Libraries were subjected to cluster generation on flow cell and paired-end sequencing in a MiSeq sequencer platform (Illumina). Sequence data were analyzed with the instrument software MiSeq Reporter v.2.5.1 (Illumina), and the reads were aligned against the human reference sequence GRCh37. The resulting VCF files were visualized using the VariantStudio v.3 Software (Illumina), which supplies the report of all detected sequence variants and the respective annotation. In order to select the potentially pathogenic variants, distinct filters were applied. Primarily, data were filtered using exclusion criteria: quality parameters (alt read depth <5, low variant frequency <20%, and GQX <1000), consequence (synonymous variants with no predicted effects in splicing), variant location (downstream/upstream gene variant, and deep intronic variant >20 bp from splice site), and allele frequency (≥1%). Then, the variant pathogenic potential was evaluated through *in silico* prediction (SIFT, PolyPhen, MutationTaster) and clinical database mining (LOVD, ClinVitae,

ClinVar, Varsome). Finally, following literature review and pedigree segregation studies, the variant with the highest pathogenic potential was prioritized for each proband's sample.

Sanger sequencing and mutation analysis

After PCR amplification, the PCR products were purified using a mix of Exonuclease I (Thermo Fisher Scientific, Wilmington, DE, USA) and FastAP Thermosensitive Alkaline Phosphatase (Thermo Fisher Scientific, Wilmington, DE, USA). Sequencing products were obtained using the Big Dye Terminator v1.1 Cycle Sequencing Kit (Applied Biosystems, Foster City, CA, USA) and purified using ethanol/EDTA/sodium acetate precipitation protocol. Products were analyzed in an automated sequencer (3130 Genetic Analyzer, Applied Biosystems) with the Sequence Analysis Software version 3.4.1 (Applied Biosystems), and Variant Reporter v1.0 (Applied Biosystems) was used for sequence analysis.

Protein expression and purification

To produce recombinant CHK2 (a truncated version comprising residues 70-512, according to Cai *et al.* [45]), the synthetic gene, codon optimized for expression in *Escherichia* (*E.*) *coli*, was cloned into pET28a (ProteoGenix, Schiltigheim, France). The gene includes the sequences encoding an N-terminal 6×His tag followed by a 3C protease cleavage site. For optimization of protein production, cell growth conditions were tested regarding *E. coli* host strain and growth media. Expression conditions were selected based on SDS-PAGE analysis. Accordingly, transformed *E. coli* BL21 (DE3) Rosetta 2 cells expressing WT CHK2, p.E321A or p.I157T variants were cultured at 37 °C in Terrific Broth medium. Protein expression was induced at an OD_{600} of 2 by addition of 500 µM IPTG and proceeded for ~18 h at 18 °C. The cells were harvested by centrifugation at 8,000×g for 15 minutes, at 4 °C. The obtained pellet was disrupted in lysis buffer [50 mM sodium phosphate pH 7.5, 250 mM NaCl, 0.5 mM TCEP, 5% glycerol (v/v), 2 mM $MgCl_2$], supplemented with EDTA-free protease inhibitor cocktail tablet (Roche, Basel, Switzerland) and benzonase (Novagen, Darmstadt, Germany), in a Z Basic cell disruptor (Constant Cell Disruption Systems, Northants, UK). After centrifugation at 23,000×g, 25 min, 4 °C, the filtered supernatant was applied into two tandem 5-ml HisTrap™ High-Performance

columns (GE Healthcare, USA) and His-tagged recombinant proteins were purified by immobilized metal affinity chromatography by elution with a 10 mM–1 M imidazole gradient. Fractions containing CHK2 were incubated with 3C protease (1:100 mass/mass ratio) in 14 kDa-cut-off dialysis membranes and dialyzed overnight against 2 L dialysis buffer (50 mM NaPi pH 7.5, 250 mM NaCl, 5% glycerol, 0.5 mM TCEP, 10 mM imidazole). The proteins were recovered from the dialysis membrane, centrifuged 15 min at 23,000×g, 4 °C, and injected in a 5-ml HisTrap™ High-Performance column (GE Healthcare, USA). The flow-through sample was concentrated by ultrafiltration with Amicon-30kDa Ultra-15 centrifugal filter units (Merck, Darmstadt, Germany) and further purified by size exclusion chromatography (SEC) on a HiLoad™ 16/600 Superdex™ 200 column (GE) equilibrated in SEC buffer [50 mM sodium phosphate pH 7.5, 250 mM NaCl, 0.5 mM TCEP, 5% glycerol (v/v)]. Protein purity was analyzed by SDS-PAGE, and the protein concentration determined by the Bradford assay or NanoDrop (Thermo Fisher Scientific, MA, USA). Pure proteins were aliquoted, flash frozen in liquid nitrogen, and stored at -80 °C until further usage.

CHK2 kinase activity assays

CHK2 kinase activity was assessed for WT, p.E321A, and p.I157T, using the ADP-Glo™ Kinase Assay (Promega, Madison, WI, USA), according to the manufacturer's protocol, with minor modifications. Briefly, the assay comprises three main steps: (i) the kinase reaction; (ii) the cessation of kinase reaction plus depletion of remaining ATP by the addition of the ADP-Glo™ Reagent; (iii) the conversion of ADP product into ATP for luminescence detection when the Kinase Detection Reagent is added. For the primary enzyme reaction, 5 µl of CHK2 solution (final concentration 200 nM, in 40 mM Tris, pH 7.5, 20 mM $MgCl_2$, 0.1 mg·ml^{-1} BSA), kinase substrate (CHKtide, final concentration 0.2 mg·ml^{-1}), and different ATP concentrations were added into a 384 white, opaque bottom, low-volume microplate (ProxiPlate-384 Plus, PerkinElmer, Waltham, USA) and incubated at 30°C for 1h. For each reaction, a control without enzyme was included. For detection, 5 µl of ADP-Glo™ Reagent was added to the assay wells to stop the reaction and deplete unconsumed ATP and incubated for 40 min at 23°C; followed by

the addition of 10 μl of Kinase Detection Reagent and incubated for 30 min at 23°C. Luminescence was measured in a PHERAstar FSX plate reader (BMG LABTECH, Ortenberg, Germany).

Molecular dynamics (MD) simulations

Molecular dynamics simulations were performed for the WT and both CHK2 variants (p.I157T and p.E321A). System preparation was identical for all variants: (i) experimental structures of the truncated proteins were obtained from PDB (3I6W); (ii) all missing residues and/or atoms in the PDB files were added using MODELER v10.2 [63]; (iii) overlapping side chains were fixed with Scwrl; and (iv) substitutions I157T and E321A were generated using PyMOL (http://www.Pymol.org/).

All simulations were performed with the GROMACS 2020.2 [64] package and modelled using the Amber14sb forcefield [65], alongside the TIP3P water model [66]. Each CHK2 protein structure was inserted in a truncated octahedron box filled with water molecules (considering a minimum distance of 1.0 nm between protein and box walls). The total charge of the system was neutralized with the required number of Na^+ ions, with additional Na^+ and Cl^- ions added to the solution to reach an ionic strength of 0.15 M.

The system was energy-minimized using the steepest descent method for a maximum of 50,000 steps with position restraints on the heteroatom positions by restraining them to the crystallographic coordinates using a force constant of 1,000 $kJ \cdot mol^{-1}$ in the X, Y and Z positions. Before performing the production runs, an initialization process was carried out in 4 stages of 100 ps each. Initially, all heavy-atoms were restrained using a force constant of 1,000 $kJ \cdot mol^{-1} \cdot nm^{-1}$, and at the final stage only the Cα atoms were position-restrained using the same force constant. In the first stage, the Berendsen thermostat [67] was used to initialize and maintain the simulation at 310 K, using a temperature coupling constant of 0.01 ps, without pressure control. The second stage continued to use the Berendsen thermostat [67], now with a coupling constant of 0.1 ps. The third stage introduced isotropic pressure coupling with the Berendsen barostat [67], with a coupling constant of 5.0 ps, temperature coupling continued to use the Berendsen thermostat with

a coupling constant of 0.1 ps. In the fourth stage we changed the thermostat to V-rescale [68], with a temperature coupling constant of 0.1 ps, and the barostat to Parrinello-Rahman [69] with a pressure coupling constant of 2.0 ps. Position restraints were only applied to Cα atoms.

For production simulations, conditions were the same as for the fourth stage, but without any restraints. In all cases, 2 fs integration steps were used. Long-range electrostatic interactions were treated with the PME [70,71] scheme, using a grid spacing of 0.1 nm, with cubic interpolation. The neighbor list was updated every 5 steps with a Verlet cutoff with a 0.8 nm radius. All bonds involving hydrogen atoms were constrained using the LINCS algorithm [72].

Simulations of each system were performed for 2 μs over 3 replicates. The first 500 ns of simulation were considered as equilibration time, and the remaining frames were used for analysis. Visualization and rendering of simulation snapshots were performed with the molecular graphic viewers PyMOL (http://www.Pymol.org/) and VMD [73].

Supporting Figures

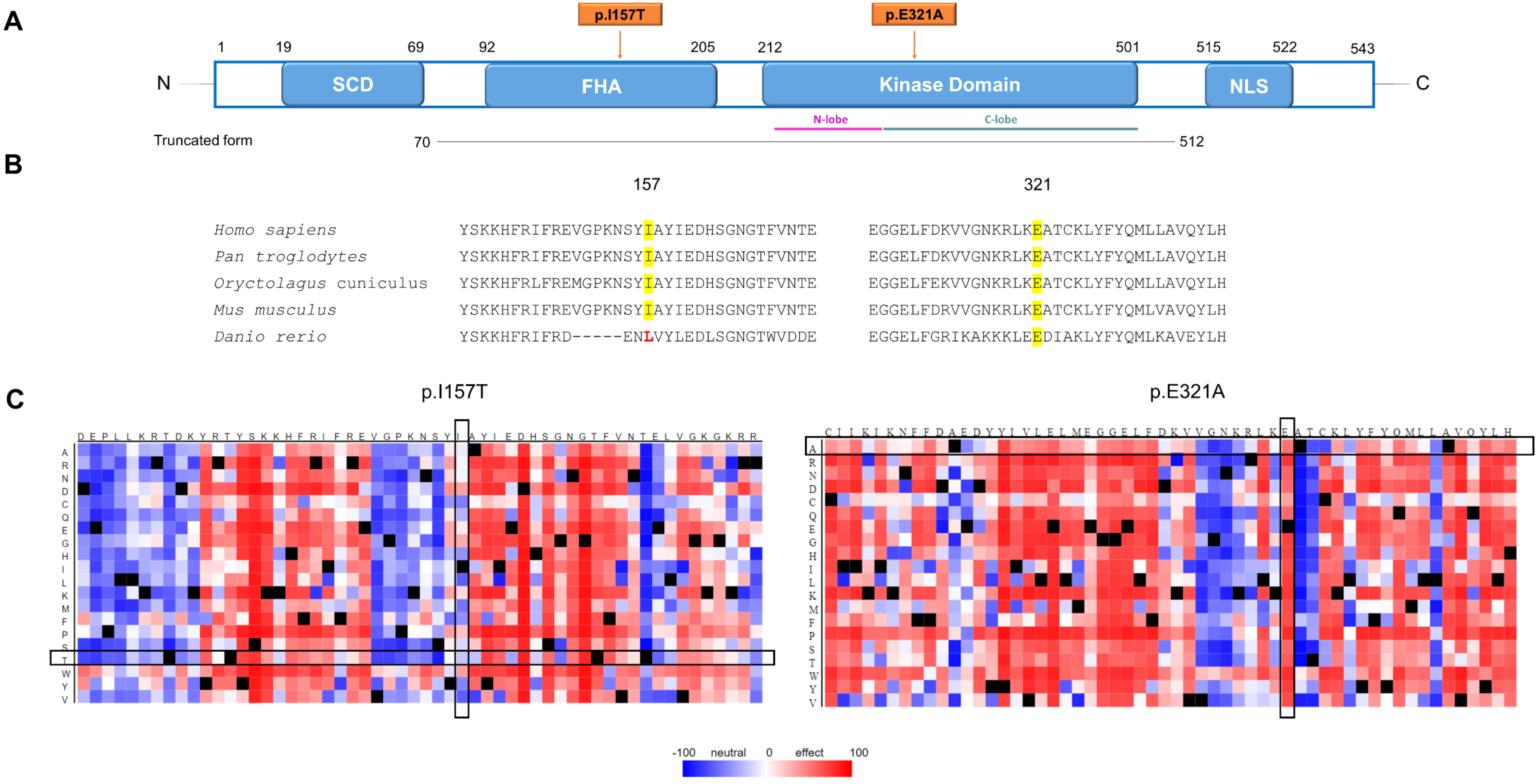

Figure S1. *In silico* studies of CHK2 proteins. (A) Linear representation of CHK2 with its domain boundaries and the truncated form, that was produced for biophysical characterization, consisting of residues 70-512 [45]. The variants identified in this study are represented in orange boxes. (B) Comparative sequence analysis across phylogeny. The mutation site is highlighted in yellow. Information retrieved from the Ensembl genome browser (https://www.ensembl.org/index.html). (C) Functional effects predicted by SNAP2 for p.I157T and p.E321A variants. Blue color indicates a low score, a variant that is predicted to have a neutral/no effect on protein structure, while red indicates a high score, for variants that are predicted to change native protein structure. SCD, SQ/TQ cluster domain; FHA, forkhead-associated domain; NLS, nuclear localization signal.

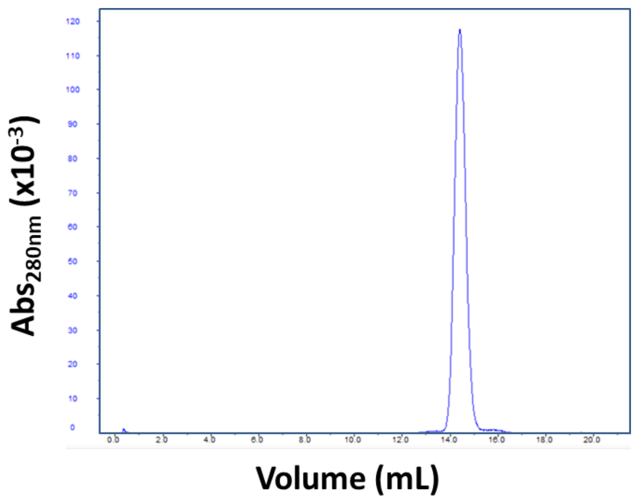

Figure S2. Analytical size exclusion chromatography to analyze the oligomeric arrangement of WT CHK2. CHK2 WT protein (100 µg) was injected at 0.5 ml/min in a S200 Increase 10/30 GL previously equilibrated and eluted with 50 mM NaPi pH 7.5, 250 mM NaCl, 10% Glycerol, 0.5 mM TCEP. CHK2 eluted at 14.4 ml, corresponding to a molecular weight of 64.6 kDa. Similar chromatograms were obtained for the CHK2 p.I157T and p.E321A variants.

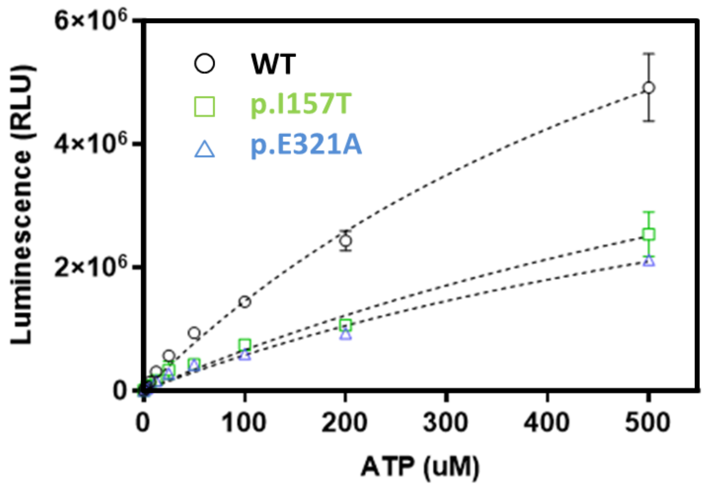

Figure S3. CHK2 kinase activity. Enzymatic activity of CHK2 WT, p.I157T, and p.E321A was measured with the ADP-Glo™ Kinase activity kit, employing a surrogate CDC25 peptide as co-substrate. Luminescence measured in relative light units (RLU) is expressed as a function of ATP concentration. At saturating ATP, the CHK2 p.I157T and p.E321A variants presented 40-50% of the WT CHK2 enzymatic activity. Two independent assays were performed, in triplicate; error bars indicate the mean ± SD.

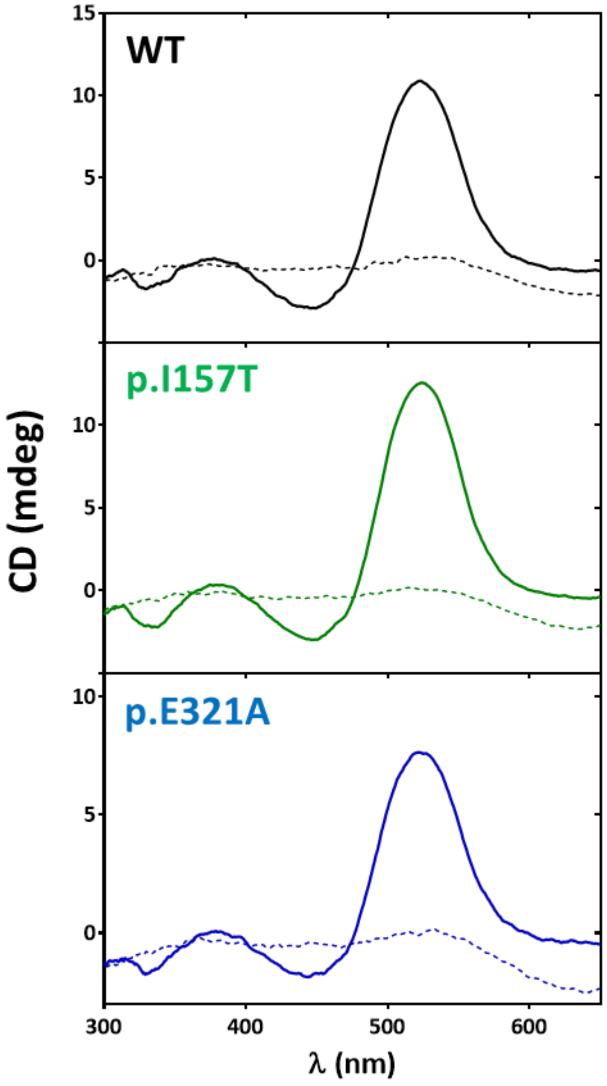

Figure S4. Induced circular dichroism confirms formation of amyloid fibrils upon CHK2 aggregation. Reaction of fibrils with Congo red was analyzed at 42 °C by induced CD, by mixing in a dual-chamber cuvette CHK2 variants at 0.2 mg·ml^{-1} (in SEC buffer) and 80 µM Congo red (in SEC buffer). Dashed lines, spectrum taken with both solutions in their segregated individual chambers prior to mixing; full lines, spectra after 1h incubation of CHK2 proteins with Congo red. Replicates from two independent protein batches were performed.

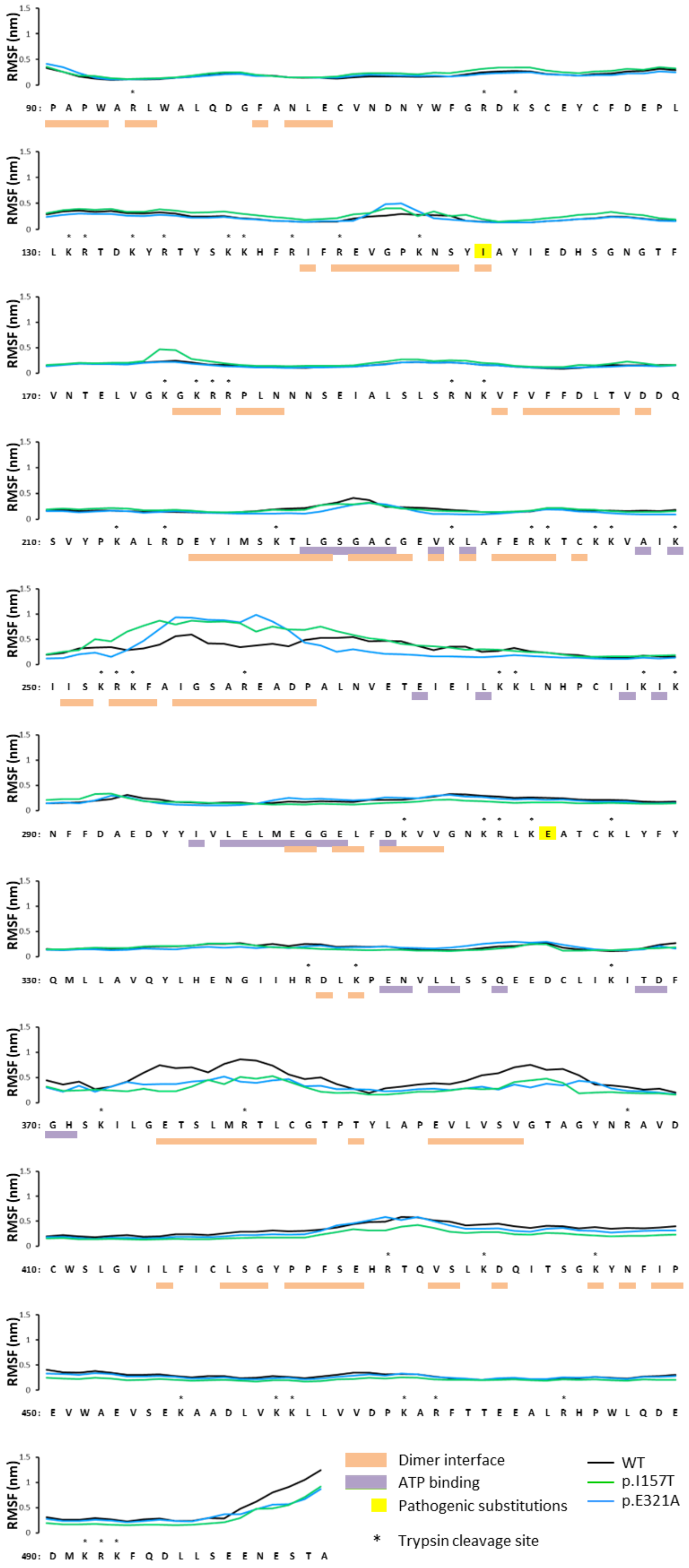
RMSF (nm)
Dimer interface
ATP binding
Pathogenic substitutions
* Trypsin cleavage site
WT
p.I157T
p.E321A

Figure S5. Structural flexibility of CHK2 variants predicted by molecular dynamics simulations. Structural flexibility expressed as root mean square fluctuation (RMSF) of WT (black line), p.I157T (green line), and p.E321A (blue line) CHK2, along the primary sequence of WT CHK2, highlighting with yellow squares the substituted residues in the protein variants. Monomer-monomer interface residues are shown with light orange boxes, residues lining the ATP binding site are shown with purple boxes, and the predicted trypsin cleavage sites are marked with asterisks (*).

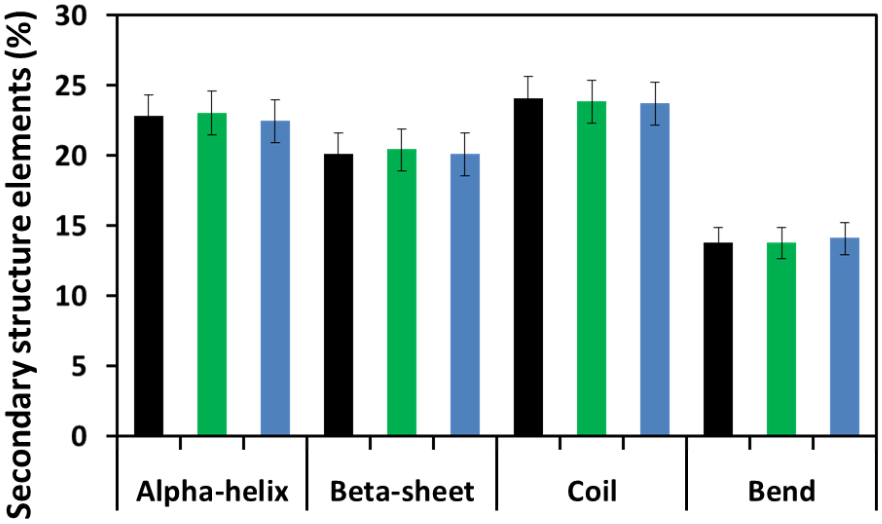

Figure S6. Secondary structure of CHK2 WT and both variants simulated. Percentage of α-helix, β-sheet, coil, and bend secondary structure elements was obtained using the GROMACS tool gmx do_dssp [64].

Supporting Tables

Table S1. Results of the immunohistochemical staining of patient's thyroid tumors and normal tissues and correlation with *CHEK2* variants' status.

CHEK2 variant	Tissue type	Immunohistochemical staining pattern			
		Negative	Positive		
			Weak	Moderate	Strong
**F24: p.I157T**					
II.1 (heteroz.)	T				Diffuse
	N		Focal		
II.6 (heteroz.)	T				Diffuse
	N				Focal
**F83: p.E321A**					
II.2 (heteroz.)	T			Focal	
	N		Focal		
II.3 (heteroz.)	T				Diffuse
	N			Focal	
: p.Y156					
III.1 (homoz.)	T	Negative			
	N	Negative			
III.3 (heteroz.)	T				Focal
	N		Focal		
III.6 (heteroz.)	T				Focal
	N		Focal		
**Wild type**					
Patient 1	T			Focal	
	N	Negative			
Patient 2	T			Focal	
	N	Negative			
Patient 3	T		Focal		
	N			Focal	
Patient 4	T		Focal		
	N		Focal		
Patient 5	T				Diffuse
	N				Focal

* refers to the family described in [23], harboring the *CHEK2* truncating mutation p.Y156*. T, tumor tissue; N, normal tissue; heteroz., variant in heterozygosity; homoz., variant in homozygosity.
